# Hyperbaric oxygen and steroids for preventing stricture after large esophageal endoscopic submucosal dissection

**DOI:** 10.1055/a-2637-1928

**Published:** 2025-08-06

**Authors:** Haotian Zeng, Jiaxing Yang, Ximin Lin, Zhenglei Xu

**Affiliations:** 1 12387 Gastroenterology, Shenzhen People's Hospital, Shenzhen, China

**Keywords:** Endoscopy Upper GI Tract, Benign strictures, Endoscopic resection (ESD, EMRc, ...), Dilation, injection, stenting

## Abstract

**Background and study aims:**

Treatment of esophageal mucosal lesions by endoscopic submucosal dissection (ESD) may lead to the formation of esophageal strictures. This trial was designed to clarify efficacy of hyperbaric oxygen therapy (HBOT)-assisted steroids in preventing postoperative strictures after ESD for large and long-segment esophageal mucosal lesions.

**Patients and methods:**

Between October 2020 and July 2023, patients who underwent esophageal ESD with a remained mucosal defect of more than three-quarters of the esophageal circumference and longer than 50 mm in diameter were retrospectively analyzed. Patients in the control group were administered one injection of triamcinolone acetonide in the submucosal layer and oral prednisone, whereas patients in the experimental group underwent HBOT along with the abovementioned steroid therapy. Furthermore, differences in postoperative stricture incidence and related adverse events between the two groups were evaluated.

**Results:**

A total of 35 patients were included in this study. Patients in the experimental group had a significantly lower stricture incidence compared with those in the control group (6.7%, 1/15 patients vs 40%, 8/20 patients; *P* = 0.048). Stricture incidence of circumferential mucosal defects was significantly lower in the experimental group than in the control group (0.0%, 0/6 patients vs 71.4%, 5/7 patients; *P* = 0.021). Incidence of post-ESD complications was similar in both groups (6.7%, 1/15 patients vs 25%, 5/20 patients; *P* = 0.207). No HBOT-related AEs were observed.

**Conclusions:**

HBOT-assisted steroid therapy might be a safe and effective way to prevent postoperative strictures after ESD for large and long-segment esophageal mucosal lesions.

## Introduction

Esophageal cancer is a highly prevalent malignant tumor of the digestive tract globally. Superficial esophageal squamous cell carcinoma (SESCC), a subtype of esophageal cancer, is characterized by lesions limited to the superficial mucosal layer and submucosal layer, with or without lymph node metastasis 1 . Esophageal intraepithelial neoplasia is a precancerous lesion. Early diagnosis and treatment can prevent onset and progression of esophageal cancer. Endoscopic submucosal dissection (ESD) is the first choice for treatment of SESCC and precancerous lesions. ESD is a minimally invasive procedure that can allow resection of an entire lesion and has long-term efficacy comparable to surgery 2 . However, large and long segmental mucosal defects after ESD can lead to formation of esophageal stricture, which considerably affects patient prognosis. Statistical studies suggest that mucosal defects of three-quarters or more of the esophageal circumference, longitudinal diameters of mucosal defects ≥ 30 to 50 mm, and infiltration of the lesion beyond the lamina propria mucosa (M2) are independent risk factors for development of strictures 3 4 5 . If no prophylactic measures are taken after ESD, incidence of stricture in mucosal defects of three-quarters or more of the circumference of the esophageal ring can increase from approximately 66% to 100% 1 , and incidence of stricture in esophageal circumference lesions can even reach 100% 1 6 7 . Other risk factors for postoperative stricture include lesion location in the cervical and upper thoracic segments, intraoperative injury to the muscularis propria, and surgical duration 5 . Furthermore, esophageal strictures are associated with tumor regression and can occur during conformal radiation therapy (CRT) 8 .

Steroids are the most commonly used prophylactic agents to prevent formation of postoperative strictures, which can be administered via endoscopic local injection and oral administration and as steroid gels 9 . However, the effectiveness of steroids in preventing stricture with post-ESD mucosal defects involving the entire circumference of the esophagus is unsatisfactory 3 7 10 . For post-ESD mucosal defects involving the entire circumference, incidence of postoperative stricture in patients receiving local injections of triamcinolone acetonide and oral prednisone prophylaxis regimens can range from approximately 36.4% to 81.8% 3 6 7 10 .

Hyperbaric oxygen treatment (HBOT) has the following benefits: 1) improving the hypoxic state of the peri wound tissues; 2) promoting vascularization and regeneration of epithelial cells; and 3) accelerating mucosal defect healing and inhibiting scar formation 11 12 13 14 . HBOT is a noninvasive technique to promote wound healing, which has been approved in several countries 15 . Based on the abovementioned data, this study innovatively used HBOT-assisted steroid administration to prevent stricture formation after ESD for large and long segmental esophageal mucosal lesions. Moreover, safety and efficacy of this prophylactic measure were evaluated.

## Patients and methods

### Inclusion process for participants

Data from patients who underwent ESD for esophageal intraepithelial neoplasia or SESCC at Shenzhen People’s Hospital between October 2020 and July 2023 were retrospectively collected. Inclusion criteria for this study were: 1) patient age between 18 and 80 years, irrespective of sex; 2) postoperative pathological confirmation of esophageal intraepithelial neoplasia or SESCC; 3) undergoing ESD treatment; and 4) large and long segmental esophageal mucosal defects after ESD. Large segmental esophageal mucosal were defined as postoperative traumatic mucosal defects of three-quarters or more of the esophageal circumference. Long segmental esophageal lesions were defined as postoperative mucosal defects with a longitudinal diameter ≥ 50 mm.

Exclusion criteria were: 1) presence of contraindications to use of steroids; 2) presence of HBOT; 3) previous invasive treatments, such as ESD, EMR, CRT, or endoscopic dilatation, in the region where the current ESD treatment was performed; and 4) patients who were lost to postoperative visits. The following data were collected from the study population: sex, age, lesion site, infiltration depth, postoperative mucosal defect circumference and longitudinal diameter, whole resection rate, complete resection rate, and histologic diagnosis.

Contraindications to HBOT include: 1) concurrent administration of doxorubicin, cisplatin, or disulfiram; 2) premature infants; and 3) untreated pneumothorax.

This retrospective study was performed in accordance with the ethical standards of the 1975 Declaration of Helsinki. All data were collected from medical records. Patients provided written informed consent before the procedures were performed.

### Treatment

#### ESD treatment

All patients underwent tracheal intubation and received intravenous general anesthesia in the operating room. Endoscopic ESD was performed by an experienced associate chief physician or a general chief physician (ESD experience > 500 ESDs).

Patients were monitored postoperatively for complications, such as delayed postoperative bleeding, perforation, and infection. Bleeding was defined as presence of symptoms, such as hematemesis, melena, or a drop in hemoglobin ≥ 20 g/L within 30 days after surgery. Perforation was defined as presence of subcutaneous emphysema in the neck or chest of the patient, as evidenced by physical examination or chest imaging. Infection was defined as development of signs and symptoms of infection (such as chills or fever) within 48 hours after ESD, as evidenced by laboratory tests (increased white blood cells, C-reactive protein, and procalcitonin).

#### Prevention of postoperative stricture

**Control group**

After ESD, patients were administered triamcinolone acetonide solution by multisite shallow injections into the submucosal layer of the treated wound remnant to prevent stricture 1 16 . Triamcinolone acetonide (Kenacort; 50 mg/5 mL; Bristol-Meyers Squibb, Japan) was diluted to 5 mg/mL using saline, and dosage of triamcinolone acetonide was determined based on the approximate area of the mucosal defect of 1 mg/cm ^2^ (area = length × width) 16 17 . Damage to the intrinsic muscle layer was avoided during the surgery. Patients were administered oral prednisone on postoperative Day 2 at a dose of 30 mg/day. In patients with mucosal defects that were three-quarters or more of esophageal circumference, the dose was gradually decreased by 5 mg every 2 weeks and discontinued after 12 weeks. In patients with mucosal defects involving the entire circumference, the dose was gradually decreased by 5 mg every 3 weeks and discontinued after 18 weeks 18 19 20 . During oral steroid administration, patients were orally administered two tablets of compounded calcium carbonate D3 chewable tablets (Caltrate, each tablet contains 300 mg of calcium and 60 IU of vitamin D3, Wyeth, United States) daily to prevent osteoporosis. Patients were also administered the oral proton pump inhibitor rabeprazole (Pariet, 10 mg/tablet, Eisai, Japan) and aluminum phosphate gel (Phosgel, 20 g/bag, Boryung Pharmaceutical Co. Ltd, Japan) to protect the postoperative condition of the esophagus 21 .

**Experimental group**

**Fig. 1:**
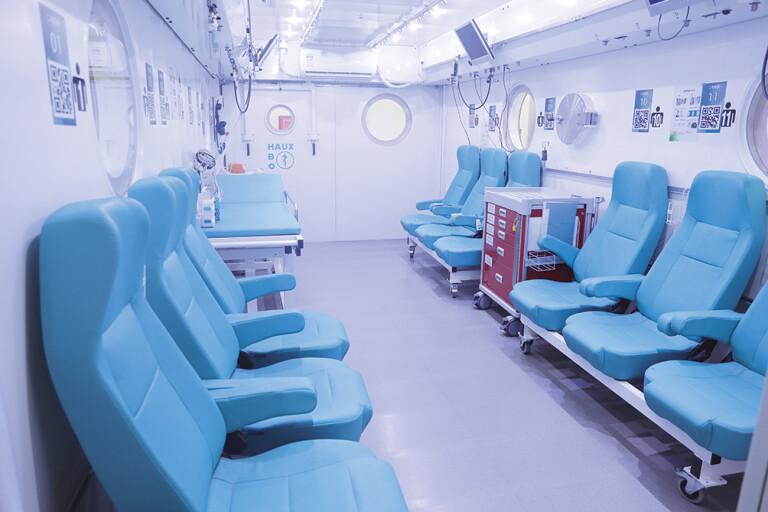
Medical air pressurized oxygen chamber.

Patients in the experimental group were treated with the same medication regimen as those in the control group. After exclusion of contraindications to HBOT, patients underwent HBOT on postoperative Day 2. A medical air pressurized oxygen chamber (STARMED-QUADOR; HAUX, Germany) was used for the primary supply of oxygen ( Fig. 1 ), with a therapeutic pressure of 0.2 MPa (2.0 ATA), 10 minutes of ramping up, 20 minutes of steady-pressure oxygen inhalation thrice, and 10 minutes of decompression to ordinary pressure to exit the chamber (once/day). In total, patients underwent 20 treatment sessions over 8 weeks. Each patient was scheduled for two to five treatment sessions per week based on their personal schedule. Patients were monitored for intraoperative and postoperative HBOT-associated adverse reactions.

**Follow-up visits**

Gastroscopy was performed after ESD at weeks 3, 6, and 12. If there was no stricture at the 12-week follow-up visit, the patient was instructed to undergo reexamination after 6 to 12 months to evaluate healing of the mucosal defect, presence of stricture, and recurrence. Patients were followed up by telephone at 1, 3, 6, and 12 months after surgery to inquire about any discomfort, including dysphagia, and were instructed to undergo gastroscopy at any time if symptoms of dysphagia appeared to observe for presence of any esophageal stricture. Furthermore, patients found to have esophageal stricture during the follow-up period were treated with endoscopic balloon dilatation (EBD). Esophageal stricture was defined as difficulty in passing the narrowest part of the esophagus with a standard endoscope (GIF-Q260J; Olympus Medical Systems, Tokyo, Japan) 6 16 . Refractory stricture was defined as failure to pass the stricture site via standard endoscopy or re-diagnosis of esophageal stricture after more than three treatment sessions of EBD 3 4 . Time of stricture occurrence was defined as time when the stricture was confirmed via endoscopy. The Stooler’s dysphagia classification was used as a secondary evaluation index 16 . Physical examination and blood tests were performed at each regular endoscopic reexamination to assess the adverse effects of steroids. All patients were followed up until November 2023.

**Outcome indicators**

Esophageal stricture formation after ESD was considered the primary endpoint of this study. Number of endoscopic stricture treatment sessions, incidence of refractory strictures, other postoperative complications, including bleeding, perforation, and infection, and stricture prevention-associated adverse events (AEs) were considered the secondary endpoints. Steroid steroid-associated AEs included delayed perforation after local injection of steroids and systemic AEs included diabetes mellitus, peptic ulcers, osteoporosis, steroid-related psychiatric disorders, and edema after oral administration of steroids. HBOT-associated AEs included oxygen toxicity, barotrauma, and decompression disease.

#### Statistical analysis

Statistical analysis was performed using SPSS 25.0 software. Measurement data were evaluated for normal distribution using the Shapiro–Wilk test. A *t* -test was performed for comparison between two groups of normally distributed measurement data. Wilcoxon rank-sum test was performed for measurement data conforming to skewed distribution. Fisher’s exact test was performed for comparison of enumeration data. For all tests, a two-tailed *P* -value of <0.05 was considered statistically significant.

## Results

### Comparison of baseline characteristics

**Fig. 2:**
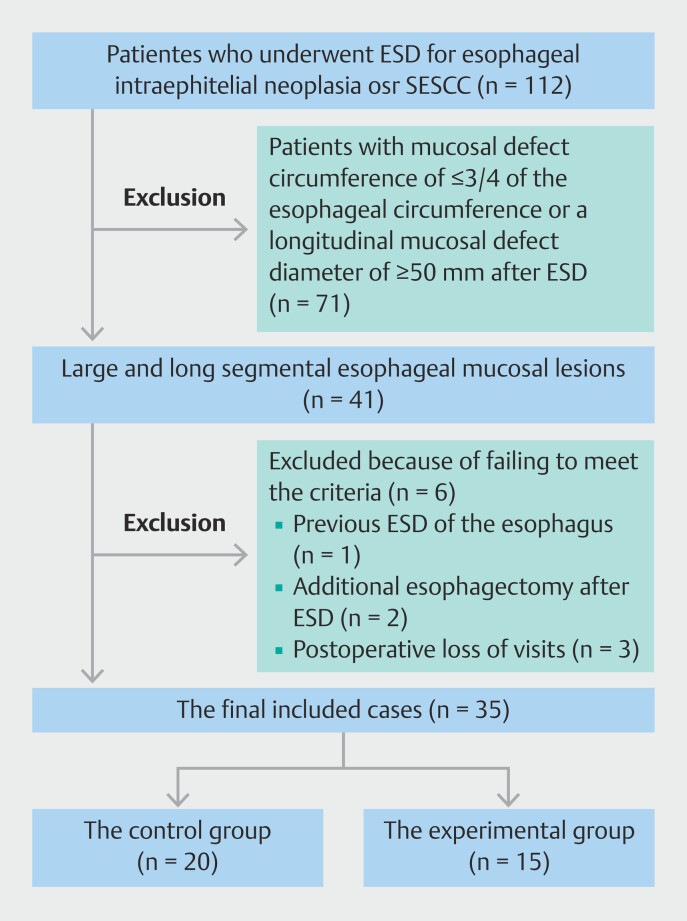
Overview of patient inclusion process. ESD, endoscopic submucosal dissection; HBOT, hyperbaric oxygen treatment.

A total of 112 patients who underwent ESD at Shenzhen People’s Hospital between October 2020 and July 2023 for esophageal mucosal lesions were included in this study. Among them, 41 patients (36.6%) met the definition of large and long segmental lesions of the esophagus. Six patients were excluded due to previous ESD of the esophagus (n = 1), additional esophagectomy after ESD (n = 2), and postoperative loss of visits (n = 3). Eventually, 35 patients were included in this study. Among them, 15 patients received HBOT combined with steroid therapy and were included in the experimental group, whereas the other 20 patients who received only steroid therapy were included in the control group ( Fig. 2 ). No significant difference was observed between the two groups in terms of age, gender, smoking and drinking behaviors, lesion site, depth of infiltration, postoperative circumference and longitudinal diameter of the mucosal defects, whole resection rate, complete resection rate, and histologic diagnosis. Longitudinal diameter of the mucosal defects was insignificantly longer in the experimental group than in the control group (median 7 cm vs 6 cm; *P* = 0.114). The postoperative mucosal defect was extended to the entire circumference of the esophagus in six (40%) and seven patients (35%) in the experimental and control groups, respectively ( *P* > 0.999). All lesions were completely resected (R0) without vascular or lymph node metastasis. One lesion in each of the two groups (6.7% vs 5.0%, *P* > 0.999) invaded the submucosa (SM1) postoperatively. Postoperative CRT was performed. In addition, a chest computed tomography scan was performed 1 year post-surgery, showing no evidence of local recurrence or distant metastasis ( Table 1 ).

**Table:** **Table 1** Baseline patient and tumor characteristics in both groups.

Characteristic	Control group (n = 20)	Experimental group (n = 15)	*P* value
**Age (mean ± SD, year)**	62.25 ± 7.71	63.33 ± 5.78	0.651
**Sex, n (%)**			
Male	13 (65.0)	10 (66.7)	
Female	7 (35.0)	5 (33.3)	> 0.999
Smoking	5 (25.0)	5 (33.3)	0.712
Drinking	6 (30.0)	1 (6.7)	0.199
**Lesion location, n (%)**			
Cervical esophagus	0 (0)	0 (0)	> 0.999
Upper thoracic esophagus	3 (15.0)	2 (13.3)	> 0.999
Mid-thoracic esophagus	8 (40.0)	5 (33.3)	0.737
Lower thoracic esophagus	9 (45.0)	8 (53.3)	0.738
**Postoperative pathology, n (%)**			
LGIN	0 (0)	3 (20.0)	0.070
HGIN/carcinoma in situ	13 (65.0)	9 (60.0)	> 0.999
ESCC	7 (35.0)	3 (20.0)	0.458
**Extent of defect after ESD, n (%)**			
3/4	3 (15.0)	2 (13.3)	> 0.999
4/5	10 (50.0)	7 (46.7)	> 0.999
Full circumference	7 (35.0)	6 (40.0)	> 0.999
**Longitudinal extension of post-ESD mucosal defect, mean (range), cm**	6 (5–9)	7 (5–11)	0.114
**Depth of infiltration, n (%)**			
M1~M3	19 (95.0)	14 (93.3)	
SM1	1 (5.0)	1 (6.7)	> 0.999
**En bloc resection rate, n (%)**	20 (100)	15 (100)	> 0.999
**R0 resection rate, n (%)**	20 (100)	15 (100)	> 0.999
**CRT, n (%)**	1 (5.0	1 (6.7)	> 0.999
**Follow-up period, mean ± SD, month**	24.55 ± 9.48	16.20 ± 9.10	0.013
**Local recurrence, n (%)**	0 (0.0)	0 (0.0)	> 0.999
M1, lesion confined to the mucosal epithelium layer. M2, lesion invading the mucosal lamina propria. M3, lesion invading the muscularis mucosae. SM1, lesion infiltrating into the submucosal layer (< 200 μm of submucosa). Complete resection (RO), excision of the lesion in its entirety, with no tumor formation detected histologically at the vertical and lateral margins. CRT, conformal radiation therapy; ESCC, esophageal squamous cell carcinoma; ESD, endoscopic submucosal dissection; HGIN, high-grade intraepithelial neoplasia; LGIN, low-grade intraepithelial neoplasia; SD, standard deviation.			

### Safety and feasibility of prevention regimens

**Fig. 3:**
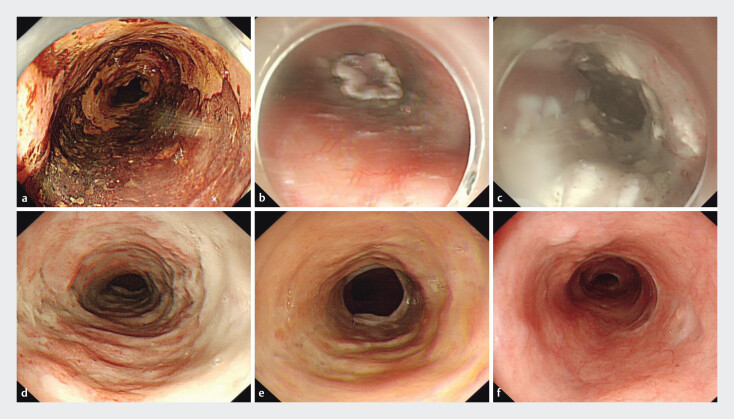
Representative case. A 56-year-old male patient underwent endoscopic submucosal dissection (ESD) for a large and long segmental superficial esophageal squamous cell carcinoma. **a** A faintly stained area was visible 28 to 38 cm from the incisors as shown by iodine-stained pigmented endoscopy, involving three-quarters of the esophageal circumference to the periphery. **b** Post-ESD mucosal defect involving the entire circumference of the esophagus lumen, with a longitudinal extension of 11 cm. **c** After ESD, the patient was injected endoscopically with the triamcinolone acetonide solution into the residual submucosa. He started oral prednisone and hyperbaric oxygen therapy on postoperative Day 2. **d** Endoscopic examination at 3 weeks postoperatively suggested good proliferation of granulation tissue in the treated wound and smooth endoscopic passage. **e** Endoscopic examination at 3 months postoperatively suggested scar formation in the treated wound and no esophageal stricture. **f** Gastroscopic esophagus did not show stricture on follow-up examination at 6 months postoperatively.

Incidence of postoperative esophageal stricture after ESD was significantly lower in the experimental group (6.7%, 1/15 patients) than in the control group (40%, 8/20 patients, *P* = 0.048). Furthermore, for patients with postoperative mucosal defects of four-fifths or more of the esophageal circumference, stricture incidence was lower in the experimental group than in the control group (7.7%, 1/13 patients vs. 47.1%, 8/17 patients; *P* = 0.042). No stricture was present in patients with post-ESD mucosal defects involving the entire circumference of the esophagus in the experimental group (0/6 patients, 0.0%). Stricture incidence was significantly lower in the experimental group than in the control group (5/7 patients, 71.4%, *P* = 0.021). Furthermore, no significant difference was observed between the two groups in terms of the number of stricture treatment sessions ( *P* = 0.778), refractory stricture incidence ( *P* = 0.244), or time to stricture ( *P* = 0.444). In each group, one patient (6.7% vs. 5.0%, *P* > 0.999) experienced delayed bleeding after ESD, which disappeared after endoscopic treatment. Four patients (20% vs 0%, *P* = 0.119) in the control group developed postoperative pulmonary infection, which improved after anti-infective treatment. During long-term follow-up, steroid-related complications, such as facial puffiness, were observed (13.3% 2/15 patients vs 15.0% 3/20 patients, *P* > 0.999), which disappeared after discontinuation of the steroid therapy. No HBOT-associated AEs were observed. In addition, no local recurrence, metastatic tumor, or treatment-related deaths were recorded ( Table 2 ). Fig. 3 shows a representative case of a patient who underwent ESD for large and long segmental SESCC.

**Table:** **Table 2** Main outcomes and other adverse events in both groups.

Characteristic	Control group (n = 20)	Experimental group (n = 15)	*P* value
**Frequency of stricture, n (%)**	8 (40.0)	1 (6.7)	0.048
**Frequency of refractory stricture, n (%)**	3 (15.0)	0 (0.0)	0.244
**Time to esophageal stricture, mean (range), day**	54.5 (24–124)	118	0.444
**No. required EBDs, median (range)**	2 (1–8)	1	0.778
**Stricture incidence in > 4/5 circumferential lesions**	8/17 (47.1)	1/13 (7.7)	0.042
**Stricture incidence in mucosal defects involving the entire circumference of the esophagus**	5/7 (71.4)	0/6 (0.0)	0.021
**Stooler’s dysphagia classification**			
Grade 0	12	14	0.048
Grade I	3	0	0.244
Grade II	5	1	0.207
Grade III	0	0	> 0.999
Grade IV	0	0	> 0.999
**Surgery-related AE, n (%)**			
Total postoperative complication rate	5 (25.0)	1 (6.7)	0.207
Post-ESD hemorrhage	1 (5.0)	1 (6.7)	> 0.999
Perforation	0 (0.0)	0 (0.0)	> 0.999
Pulmonary infection	4 (20.0)	0 (0.0)	0.119
**Steroid-related AE, n (%)**			
Delayed perforation	0 (0.0)	0 (0.0)	> 0.999
Newly diagnosed diabetes mellitus	0 (0.0)	0 (0.0)	> 0.999
Peptic ulcer	0 (0.0)	0 (0.0)	> 0.999
Osteoporosis	0 (0.0)	0 (0.0)	> 0.999
Corticosteroid psychosis	0 (0.0)	0 (0.0)	> 0.999
Facial puffiness	3 (15.0)	2 (13.3)	> 0.999
**HBOT-related AE, n (%)**			
Decompression disease	0 (0.0)	0 (0.0)	> 0.999
Barotrauma	0 (0.0)	0 (0.0)	> 0.999
Oxygen toxicity	0 (0.0)	0 (0.0)	> 0.999
AE, adverse event; EBD, endoscopic balloon dilation; ESD, endoscopic submucosal dissection; HBOT, hyperbaric oxygen treatment.			

## Discussion

This exploratory study is the first to employ the HBOT-assisted steroid regimen to prevent postoperative strictures in large and long segmental esophageal mucosal lesions. This optimized regimen ensures both patient safety and treatment efficacy and possesses high application potential.

Postoperative mucosal defects undergo four pathological stages to form esophageal strictures, namely acute inflammatory response, angiogenesis, cell migration and proliferation, and tissue remodeling, to form strictures 22 . Fibrosis of the submucosal layer and the muscularis propria is currently considered a key factor causing esophageal stricture after ESD 22 23 . Steroids reduce local inflammatory responses and delay tissue fibrosis 24 ; however, efficacy of steroid monotherapy regimens is limited for circumferential ESD. Local injection of triamcinolone acetonide has resulted in a postoperative stricture incidence range of 80% to 100% for circumferential ESD, whereas incidence in patients receiving oral prednisone administration ranges from 27.3% to 100% 7 10 . A study revealed that the long-term regimen (30 mg/d, 5 mg reduction every 3 weeks for 18 weeks) led to a significantly lower stricture incidence than that recorded for the short-term regimen (tapering over 8 weeks) (36.4% vs. 82%; *P* < 0.05) 20 . The results suggested that the long-term regimen possessed a high potential for preventing postoperative strictures after ESD for circumference. A study combined triamcinolone acetonide injections with a long-term regimen to prevent esophageal strictures after circumferential ESD. The results demonstrated a decreased stricture incidence in the combination group (18.6%, 3/16 patients) compared with the oral steroid group (33.3%, 8/24 patients) and the local steroid injection group (100.0%, 4/4 patients; *P* < 0.05) 19 .

Combining multiple approaches is a viable option for preventing postoperative strictures in post-ESD mucosal defects involving the entire circumference of the esophagus 7 . Based on the limited data available, incidence of stricture prevented by polyglycolic acid (PGA) combined with fully-covered self-expandable metallic stents (FCES) ranges from 42.9% to 66.7% 25 26 27 . In addition, stricture incidence for patients with autologous skin-grafting surgery (ASGS) combined with FCES ranges from 36.8% to 88.9% 28 29 . The present patients who received the combination therapy had lower stricture incidence and fewer stricture treatment sessions compared with those reported in previous studies ( Table 3 ) 3 6 26 28 30 31 32 33 34 .

**Table:** **Table 3** Studies on prevention of esophageal strictures formed by large mucosal defects after ESD.

Study and publication year	Study design	Methods	Stricture incidence	Number of EBDs required	Follow-up period
**Prevention of esophageal strictures due to greater than 3/4 circumferential mucosal defects after ESD surgery**					
Ezoe 30 , 2011	Retrospective	Preventive EBD	59% (17/29)	6 (3–9)	/
Sakaguchi 31 , 2015	Prospective single-arm	PGA	37.5% (3/8)	0.8 ± 1.2	/
Chai 26 , 2018	Randomized controlled trial	PGA+FCES	20.5% (7/34)	4 (2–5)	/
Wen 32 , 2014	Randomized controlled trial	FCES	18.2% (2/11)	0.45 (0–3)	3 months
Current study, 2023	Retrospective	Intralesional TA injection+ oral PDN+HBOT	6.7% (1/15)	1	16.2 ± 9.1 months
**Prevention of esophageal strictures due to post-ESD mucosal defects involving entire circumference of esophagus**					
Kadota 3 , 2020	Single-arm	Intralesional TA injection+ oral PDN	61.5% (16/26)	Median 6 (1–29)	/
Kadota 6 , 2016	Retrospective	Intralesional TA injection+ oral PDN	71.4% (10/14)	5.5 (4–8.3)	/
Yamaguchi [36], 2011	Retrospective	Preventive EBD	100% (3/3)	15.6	6 months
Chai 26 , 2018	Randomized controlled trial	PGA+FCES	42.9% (6/14)	/	/
Ye 33 , 2016	Prospective single-arm	STER+FCES	17.4% (4/23)	3(1–6)	16 months (range 5–28 months)
Liao 28 , 2018	Prospective single-arm	FCES+ASGS	88.9% (8/9)	2.7 (0–6)	16.8 months (range 4.5–23 months)
Chai 34 , 2019	Prospective single-arm	STER+FCES+ASGS	37.5% (3/8)	/	7 months (range 5–10 months)
Current study, 2023	Retrospective	Intralesional TA injection+ oral PDN+HBOT	0.0% (0/6)	0	20.5 months (4–23 months)
EBD, endoscopic balloon dilatation; ESD, endoscopic submucosal dissection; FCES, fully-covered self-expandable metallic stent; HBOT, hyperbaric oxygen treatment; PDN, prednisolone; PGA, polyglycolic acid; STER, submucosal tunneling endoscopic resection; TA, triamcinolone acetonide.					

Adequate oxygen supply is essential for wound healing. Herein, the HBOT-assisted steroid was innovatively used to prevent esophageal strictures after ESD, and satisfactory results were achieved. Postoperative stricture incidence in the HBOT combined with the steroid group was significantly lower than that in the steroid-alone group (6.7% vs. 40%, *P* < 0.05). This therapy was especially effective in patients with mucosal defects of four-fifths or more of the circumference after ESD. This group may be the true target population that will benefit most from HBOT. However, HBOT was not advantageous in significantly reducing the number of stricture treatment sessions and incidence of refractory strictures because of insufficient statistical power. Herein, the control group showed a longer postoperative follow-up than the experimental group (24.55 months ± 9.48 vs. 16.20 months ± 9.10). However, esophageal strictures usually occur within 2 to 4 weeks of ESD 7 , and all patients in the present study had a postoperative follow-up of more than 4 months. In addition, one patient in the control group underwent CRT because of pathologically confirmed lesion invasion into the submucosal layer. The stricture occurred in the patient before CRT (an esophageal stricture was detected on postoperative Day 41, no further stricture was observed after treatment with EBD, and CRT was initiated on postoperative Day 118). Furthermore, we observed that one patient in the experimental group developed esophageal stenosis. Although this patient did not present with circumferential lesions, the lesion was situated in the upper portion of the esophagus. Prior research has suggested that lesions found in the upper thoracic region may constitute a risk factor for development of strictures 5 .

HBOT may play a role in preventing esophageal strictures as follows. HBOT can increase partial pressure of tissue oxygen and oxygen diffusion distance to avoid the ischemic-hypoxic state of esophageal mucosal defects 11 14 . It maintains an appropriate immunoregulation level in the body and avoids excessive inflammatory responses. Herein, no patients with postoperative infections were included in the experimental group, whereas there were four (20%) in the control group ( *P* = 0.119), which may be attributed to the enhanced anti-infective ability of HBOT on postoperative wounds. In addition, infection control reduces venous fluid leakage, thereby reducing tissue swelling at the esophageal mucosal defect and providing a favorable environment for defect repair 11 12 13 14 . HBOT alters levels of some growth factors by regulating signaling cascade pathways, thus promoting healing of esophageal mucosal defects after ischemic and inflammatory injuries. Growth factor regulation by HBOT is not unidirectional and is adaptive to the corresponding stage at which the wound is located, thereby restoring physiological homeostasis 11 . Hypoxia-inducible transcription factor-1α (HIF-1α) plays a pivotal role in macrophage activation. In addition, it is involved in expression of vascular endothelial growth factors and stromal cell-derived factors (SDFs) 11 13 14 . During wound healing, HBOT enhances HIF-1α activation, promotes angiogenesis, inhibits inflammatory responses, and accelerates healing 12 13 14 . However, during scar proliferation, HBOT inhibits fibroblast proliferation and reduces collagen fiber synthesis and secretion by down-regulating HIF-1α, thereby reducing scar formation 35 . Moreover, HBOT downregulates inflammatory responses by affecting insulin-like growth factor-1R (IGF-1R), nitric oxide (NO)-induced growth factors, and nuclear factor κ-B (NF-κB), thereby promoting healing of esophageal mucosal defects 11 12 13 . However, this hypothesized mechanism requires further validation in animal studies.

Typically, HBOT-associated AEs are related to oxygen concentration and pressure, including oxygen toxicity, barotrauma, and decompression disease. Incidence of AEs is low and mostly self-limiting. None of the 15 patients who received HBOT had HBOT-associated AEs. Compared with invasive methods such as prophylactic EBD, PGA tablet coverage, ASGS, and FCES, HBOT reduced unnecessary procedures and injuries, guaranteed effectiveness, and reduced patient pain and treatment costs.

However, there are some limitations to this study. First, selective bias may occur because of the retrospective nature. In addition, confounding factors may exist when compared with other studies because of the different definitions of strictures and refractory strictures among different studies. Furthermore, this was a single-center study with a small sample size, and generalizability of the results is limited.

## Conclusions

This study suggests that combining HBOT with steroids may offer a clinically feasible and safe approach to preventing postoperative strictures following ESD for large, long-segment esophageal mucosal lesions. As a proof-of-concept investigation, these findings warrant validation through well-designed randomized controlled trials.
